# Climate impact dataset of 1233 ingredients to promote sustainability of food service operators in Finland

**DOI:** 10.1016/j.dib.2024.111143

**Published:** 2024-11-15

**Authors:** Kim Lindfors, Venla Kyttä, Oona Pietiläinen, Merja Saarinen, Virpi Vorne

**Affiliations:** a Natural Resources Institute Finland (Luke), Latokartanonkaari 9, 00790 Helsinki, Finland; b Natural Resources Institute Finland (Luke), Tietotie 4, 31600 Jokioinen, Finland; c Natural Resources Institute Finland (Luke), Paavo Havaksen tie 3, 90570 Oulu, Finland

**Keywords:** Life cycle assessment (LCA), Food, Sustainability, Environmental impacts

## Abstract

The food service and restaurant industry play a crucial role in promoting sustainable food consumption by offering sustainable meal options, shaping consumer preferences, and introducing eco-friendly practices. To enable the food services operating in Finland to provide more sustainable options, we created a climate impact dataset of 1233 typical ingredients used in Finnish food services. The dataset was created using Life Cycle Assessment (LCA) to assess the climate impacts of ingredients from cradle to wholesale. The climate impacts in the dataset were assessed using the characterisation factors from the IPPC's sixth assessment report, using a functional unit of 1 kg of ingredient. The final climate impacts of the ingredients include both impacts from domestic and imported products, aggregated by calculating the degree of domestic origin -weighted average. The climate impacts of Finnish plant production were assessed based on data derived from ProAgria's field plot database, and the impacts of animal and fish production were derived from recent Finnish LCA studies. The post-farm processing was assessed using data from the Agribalyse database, by changing the energy inputs of the processes to Finnish energy and modifying the transport profile to reflect Finnish conditions. The impacts of imported products were also assessed using the Agribalyse database and changing the energy inputs and transport profiles accordingly to better reflect average European production. In addition, for imported products, transportation to Finland was added. The data presented in this dataset can be utilised in other LCA studies to assess the impacts of food ingredients used in Finland in menu, or diet level assessments. As the dataset is compatible with the Finnish Food Composition Database Fineli®, it enables simultaneous assessment of nutritional value, which is crucial in achieving emission reductions without weakening the nutritional quality of food consumed.

Specifications Table

**Table:** 

Subject	Environmental Science
Specific subject area	Climate impact estimates of 1233 food ingredients consumed in Finland
Type of data	Table
Data collection	The creation of the dataset included six main steps: i) reviewing existing LCA data on domestic food production, ii) assessing production of products with no available data and updating assessments of major food crops based on data derived from ProAgria's field plot database, iii) identifying relevant production data of imported products from LCA-databases, iv) modelling the processing of agricultural products into ingredients based on an LCA database by altering raw materials and other inputs in the database, and v) deriving the final climate impacts for ingredients by calculating weighted averages based on the degree of domestic origin. vi) using category average values for missing values
Data source location	Natural Resources Institute Finland (Luke)
Data accessibility	Repository name: Climate impact dataset for the food service sector Data identification number: 10.23729/85b4539f-335a-43d3-812f-ed72f6503164 Direct URL to data: https://doi.org/10.23729/85b4539f-335a-43d3-812f-ed72f6503164 Instructions for accessing these data: Dataset downloadable as a CSV file on the Data tab.

## 1 Value of the Data

• The food system is one of the main contributors to climate change, and therefore, information on climate impacts is necessary to support the adoption of more climate-friendly food choices and practices.
• Providing data on climate impacts of food ingredients provides an opportunity for different operators, such as food services, to facilitate more sustainable food selection. These data are useful in understanding the climate impacts of food consumption in Finland and similar conditions.
• The data presented in this dataset can be used to assess the impacts of food ingredients used in Finland in menu, or diet level assessments.
• Researchers and information service providers can use the data contained in the dataset in general assessments of the climate impacts of meals and menus in Finland.
• Researchers and information service providers can use the data in general assessments of the climate impacts of diets in Finland, considering the uncertainties related to the fact that the degree of domesticity of food purchases by households differs from the purchases by food services and restaurants for some product groups.
• Researchers and information service providers can use the data in similar assessments for other countries if the uncertainties about its applicability to the conditions there are considered.

## 2 Background

The food system is one of the main contributors to climate change. The food service and restaurant industry play a pivotal role in promoting sustainable food consumption through offering sustainable meal options and shaping consumers' preferences. This dataset contributes to these efforts by providing openly available generic, ingredient-level carbon footprint data of foods. Data is tailored to the needs of the food service sector, supporting the industry's long-term carbon neutrality objectives. The dataset can be integrated into production control systems used by restaurants, which improves the usability of the data and thus supports foodservice operators in making sustainable choices in their day-to-day operations. The dataset is also compatible with the Finnish food composition database Fineli [1], enabling the assessment of climate impacts and nutrition simultaneously.

## 3 Data Description

The dataset covers 1233of the most important ingredients used in food services, covering the impacts of the entire production chain from primary production to wholesale. The dataset contains one CSV file. The name of the food ingredient is listed in Finnish, Swedish and English. Every food ingredient has an ID so that it is compatible with the Finnish food composition database Fineli [1]. The climate impact of food ingredients is expressed as both kg CO2-eq/kg and g CO2-eq/100 g.

## 4 Experimental Design, Materials and Methods

### 4.1 System definition

**Fig. 1:**
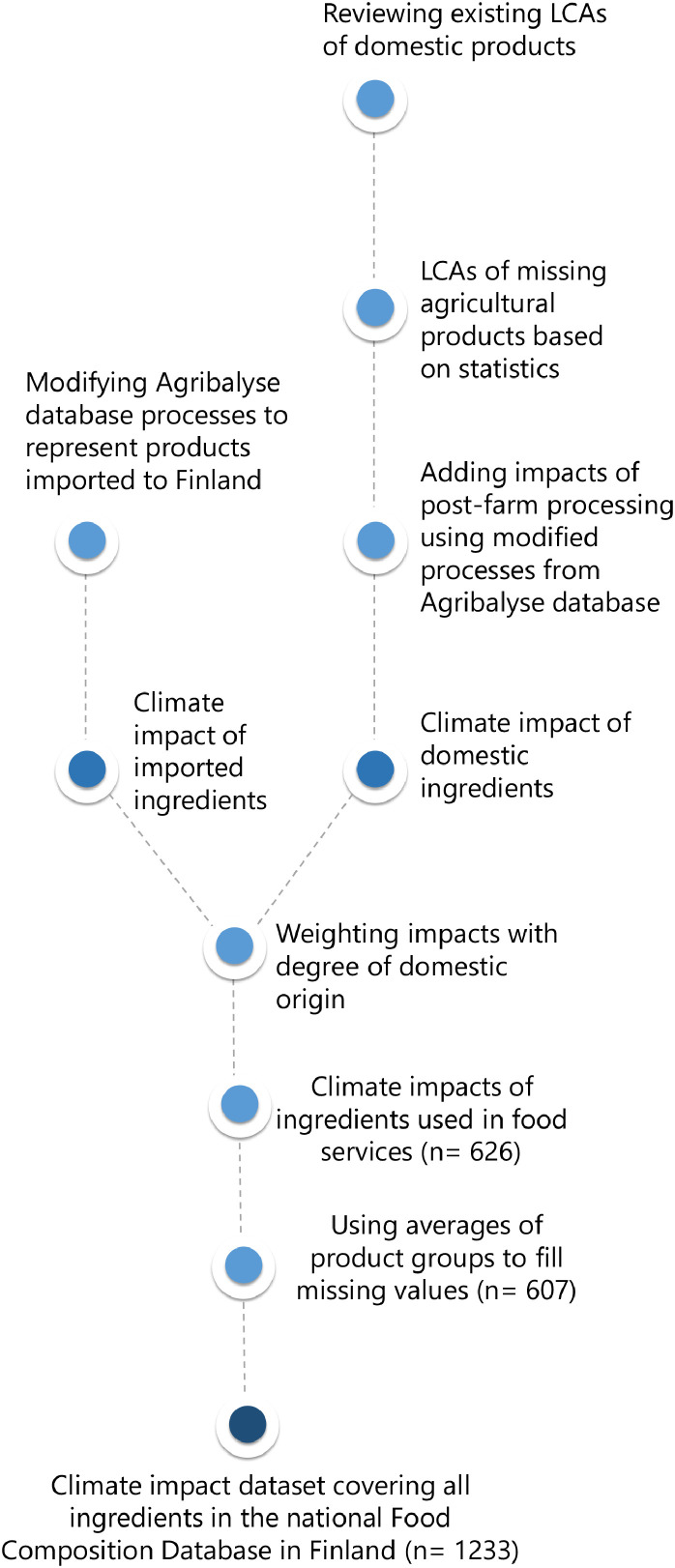
Overview on the structure of the dataset.

The climate impacts of the ingredients presented in the dataset were assessed using Life Cycle Assessment (LCA) [2]. The system boundaries of the assessment cover impacts from cradle to wholesale, and the functional unit is 1 kilogram of product. The climate impacts were assessed using the global warming potential factors provided by the IPCC's sixth assessment report [3] excluding land transformation. The dataset was created based on separate climate impact assessment of primary production and processing of Finnish and imported products, which were then aggregated into one result in the final phase by calculating the degree of domestic origin -weighted average (Fig. 1).

### 4.2 Domestic products

The climate impacts of Finnish meat, milk, and fish production were obtained from recently published LCA studies [[4], [5], [6], [7], [8]]. These studies might have different system boundaries, but the results were modified to reflect only primary production i.e. cradle-to-farmgate. The impacts of plant production were modelled based on data derived from ProAgria's field plot database, which includes information on yields, soil type, inputs such as fertiliser, plant protection, seeds, and fuel consumption. In the assessment the averages of years 2018-2022 were used. The assessment was done in SimaPro modelling software [9], where the data on agricultural inputs and their impacts were derived from Ecoinvent [13]. The soil emissions were calculated using IPCC 2006 and 2019 refined Tier 1-2 methods [10,11].

**Fig. 2:**
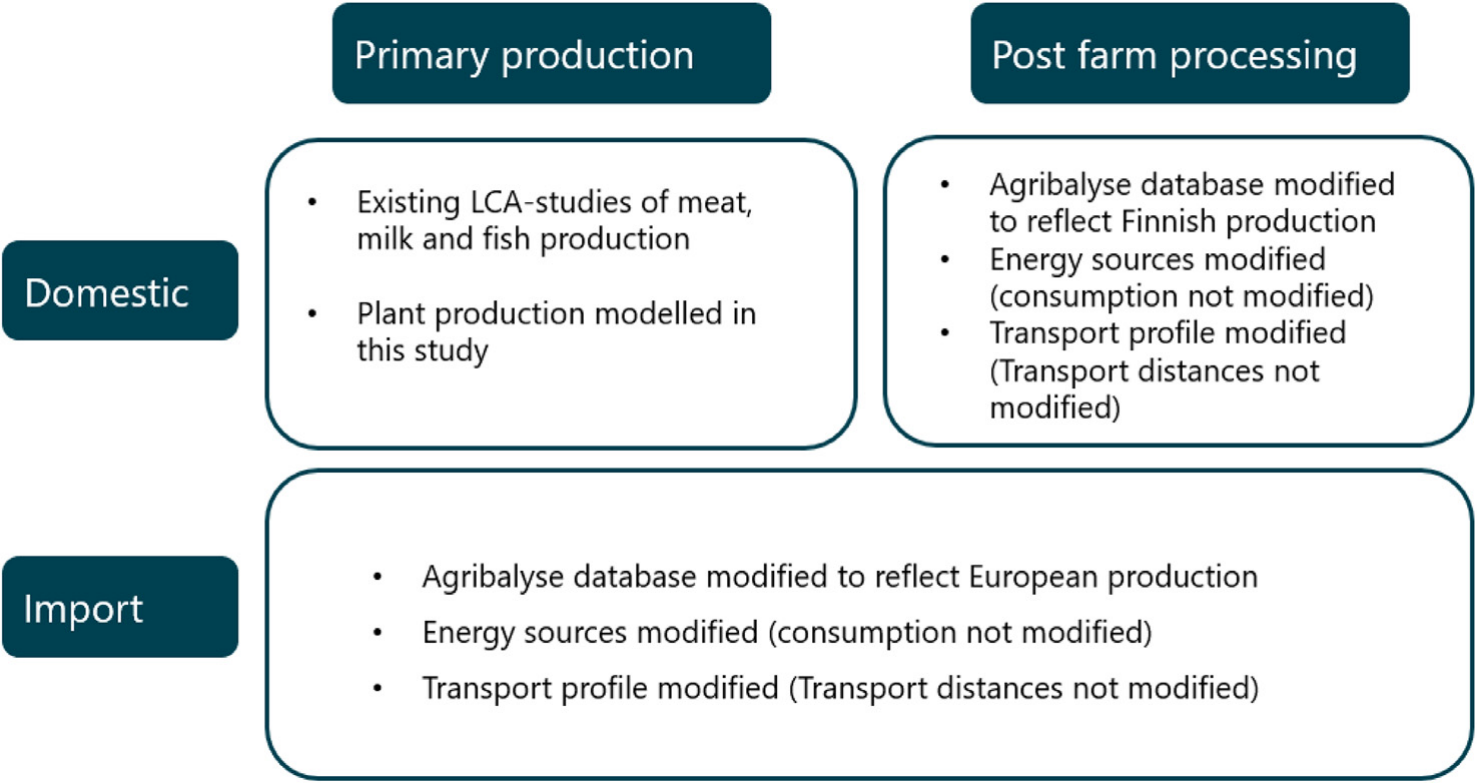
Overview of data sources.

Post farm gate processing was assessed utilising data from the Agribalyse [12] database, but the energy input of the processing was switched to a Finnish electricity mix, energy consumption was not modified as it was assumed to be representative of Finnish production. The transport profile was changed to reflect Finnish conditions; transport distances were not modified. Primary production data from Agribalyse was used, if no recently published domestic LCA study was available. Fig. 2 shows an overview of all data sources.

### 4.3 Imported products

The primary production and processing of the imported products were assessed based on the Agribalyse database [12] by switching the French energy and transport profiles to European averages. Energy sources were modified to better reflect a European average, energy consumption was left the same as it is assumed there is no major difference in production technology between locales in a European setting. Transport distances were also left the same as in the Agribalyse database, only the transport method was modified. As the Agribalyse database represents the products consumed in France, it contains the transportation of products imported to France. Therefore, transportation from central Europe to Finland was added to the products imported to Finland using data from Ecoinvent [13]. Fig. 2 shows an overview of all data sources.

### 4.4 Aggregation of impacts

The final climate impacts for products consumed in Finland were derived by calculating the weighted averages based on the degree of domestic origin of the products. This was done based on the country of origin of the primary ingredient of the product. All the products that are not cultivated in Finland at all (e.g. olive oil, nuts, exotic fruits, coffee…) were assumed to be 100 % of foreign origin.

Primarily, the degree of domestic origin of the products was calculated based on Finnish statistics that cover the domestic production and use, changes in stocks, and exports and imports of most important food commodity groupings in Finland [14]. The following formula (Formula 1) was used to calculate the degree of domestic production:

(1) Degreeofdomesticproduction:domesticproduction−exports/domesticutilisation

The statistics cover animal-based products quite comprehensively, but when it comes to other product groups like vegetables, the data is not detailed. For example, product level data is only available for tomatoes and most of the vegetable are in a group “other fresh vegetable”. In addition, in some product groups, like berries, margarins and juices, there was data missing completely and the calculations could not be done based on the statistics. In these cases, the degree of domestic origin was based on the factors used in a previous study on Finnish diets [15], and for cultivated berries, data was derived from a report covering self-sufficiency of important Finnish food products [16]. When no other data was available, the degree of domestic origin was estimated based on expert opinions.

To evaluate the accuracy of processing information in the Agribalyse database [12] in a Finnish context, the results were compared with the ones existing in previous Finnish research for processed food products.

A category level average result was used for products that did not have a direct equivalent in the Agribalyse database [12], this was done to ensure full coverage. Data quality i.e. if the result is product specific or a category average is indicated for each product.

## Limitations

The main limitations of the data pertain to its representativeness, especially regarding imported products. Since the environmental impacts of these products are assessed using a modified database on the environmental impacts of food products consumed in France, the underlying data on the countries of origin may not fully represent the countries of origin of foods consumed in Finland. This has also resulted in the challenge of quantifying the uncertainty of the data, which has not been addressed in the dataset.

The dataset does not contain impacts from land use and land-use change (LULUC). Organic products are also excluded.

The dataset is designed primarily for use by the food service sector rather than LCA practitioners. As a result, it presents only the total climate impact rather than stage-specific impacts. This simplification was made to facilitate easier integration into production control systems.

## Ethics Statement

The authors confirm that the current work does not involve human subjects, animal experiments, or any data collected from social media platforms.

## Credit Author Statement

**Kim Lindfors:** Conceptualization, Data curation, Software, Methodology, Writing – review & editing. **Venla Kyttä:** Conceptualization, Methodology, Visualization, Writing – original draft, Writing – review & editing. **Oona Pietiläinen:** Writing – original draft. **Merja Saarinen and Virpi Vorne:** Supervision, Writing – review & editing.

## Data Availability

Fairdata.fiIlmastovaikutusaineisto ruokapalvelusektorille (Original data).
